# Bis(η^5^-penta­methyl­cyclo­penta­dien­yl)aluminium tetra­bromido­aluminate

**DOI:** 10.1107/S1600536814002554

**Published:** 2014-02-08

**Authors:** Andrew P. Purdy, Cherrelle Dugger, Ray J. Butcher

**Affiliations:** a Naval Research Laboratory, Chemistry Division, Code 6100, 4555 Overlook Av, SW, Washington, DC 20375, USA; b Department of Chemistry, Howard University, 525 College Street NW, Washington, DC 20059, USA

## Abstract

The title compound, [Al(C_10_H_15_)_2_][AlBr_4_], was formed during the reduction of a mixture of Cp*AlBr_2_ and AlBr_3_. The Al^III^ atoms of the two crystallographically independent cations each lie on an inversion center, and the [AlBr_4_]^−^ anions are on general positions. At 123 K, the structure exhibits disorder in two of the Br atoms of the [AlBr_4_]^−^ ion, with a ratio occupancy of 0.733 (6): 0.267 (3). In the crystal, there is possible weak hydrogen bonding between some methyl groups and Br atoms. The interactions link the moieties in a three-dimensional array.

## Related literature   

For the tetra­chlorido­aluminate analog of the title compound, see: Macdonald *et al.* (2008 ▶); Schurko *et al.* (2002 ▶). For the formation of the Cp*_2_Al^+^ (deca­methyl­aluminocenium) cation starting from (AlCp*)_4_, see: Dohmeier *et al.* (1993 ▶); Üffing *et al.* (1998 ▶). For the formation of Cp*_2_Al^+^ starting from Cp*_2_Al*X*, see: Schurko *et al.* (2002 ▶). For other compounds containing this ion, see: Üffing *et al.* (1999 ▶); Kruczyński *et al.* (2012 ▶); Vollet *et al.* (2006 ▶); Burns *et al.* (1999 ▶). For the production of (Cp*Al)_4_ by alkali metal reduction of Cp*Al*X*_2_, see: Schormann *et al.* (2001 ▶); Minasian & Arnold (2008 ▶). For larger Al clusters containing the Cp* ligand, which have so far only been obtained from reactions between "AlX" and Cp* organometallics, see: Vollet *et al.* (2004 ▶, 2005 ▶).
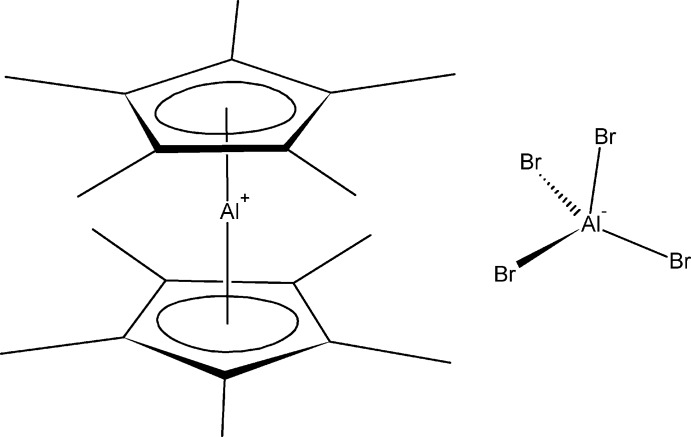


## Experimental   

### 

#### Crystal data  

[Al(C_10_H_15_)_2_][AlBr_4_]
*M* *_r_* = 644.04
Triclinic, 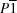

*a* = 7.8152 (4) Å
*b* = 9.2949 (4) Å
*c* = 17.5420 (8) Å
α = 85.394 (4)°
β = 85.107 (4)°
γ = 82.659 (4)°
*V* = 1256.12 (10) Å^3^
*Z* = 2
Mo *K*α radiation
μ = 6.48 mm^−1^
*T* = 123 K
0.75 × 0.28 × 0.14 mm

#### Data collection  

Agilent Xcalibur (Ruby, Gemini) diffractometer
Absorption correction: multi-scan (*CrysAlis PRO*; Agilent, 2012 ▶) *T*_min_ = 0.116, *T*_max_ = 0.506
19757 measured reflections
9539 independent reflections
5959 reflections with *I* > 2σ(*I*)
*R*_int_ = 0.038

#### Refinement  

*R*[*F* ^2^ > 2σ(*F* ^2^)] = 0.043
*wR*(*F* ^2^) = 0.072
*S* = 0.91
9539 reflections
256 parameters
24 restraints
H-atom parameters constrained
Δρ_max_ = 1.58 e Å^−3^
Δρ_min_ = −1.29 e Å^−3^

### 

Data collection: *CrysAlis PRO* (Agilent, 2012 ▶); cell refinement: *CrysAlis PRO*; data reduction: *CrysAlis PRO*; program(s) used to solve structure: *SHELXS2013* (Sheldrick, 2008 ▶); program(s) used to refine structure: *SHELXL2013* (Sheldrick, 2008 ▶); molecular graphics: *SHELXTL* (Sheldrick, 2008 ▶); software used to prepare material for publication: *SHELXTL*.

## Supplementary Material

Crystal structure: contains datablock(s) I, global. DOI: 10.1107/S1600536814002554/bh2490sup1.cif

Structure factors: contains datablock(s) I. DOI: 10.1107/S1600536814002554/bh2490Isup2.hkl

CCDC reference:

Additional supporting information:  crystallographic information; 3D view; checkCIF report

## Figures and Tables

**Table 1:** Hydrogen-bond geometry (Å, °)

*D*—H⋯*A*	*D*—H	H⋯*A*	*D*⋯*A*	*D*—H⋯*A*
C7*A*—H7*AA*⋯Br3*B* ^i^	0.98	3.03	3.886 (4)	146
C9*A*—H9*AC*⋯Br3*A*	0.98	3.04	3.773 (8)	133
C10*A*—H10*E*⋯Br3*A*	0.98	3.05	4.007 (7)	165
C9*B*—H9*BB*⋯Br1	0.98	2.95	3.770 (3)	141

Symmetry code: (i) 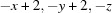.

**Fig. 1.**
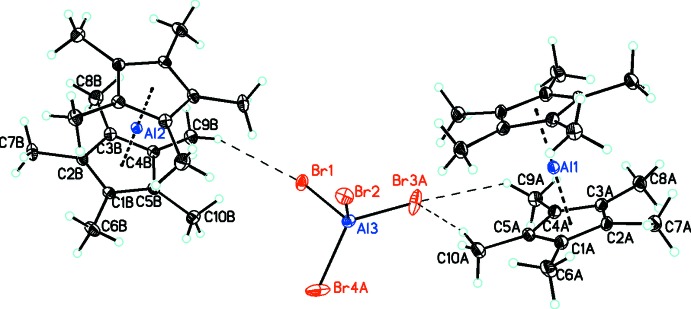
Structure of the title compound. Both nonequivalent decamethylaluminocenium cations placed on inversion centers are shown. Disordered atoms Br3b and Br4b have been omitted for clarity.

**Fig. 2.**
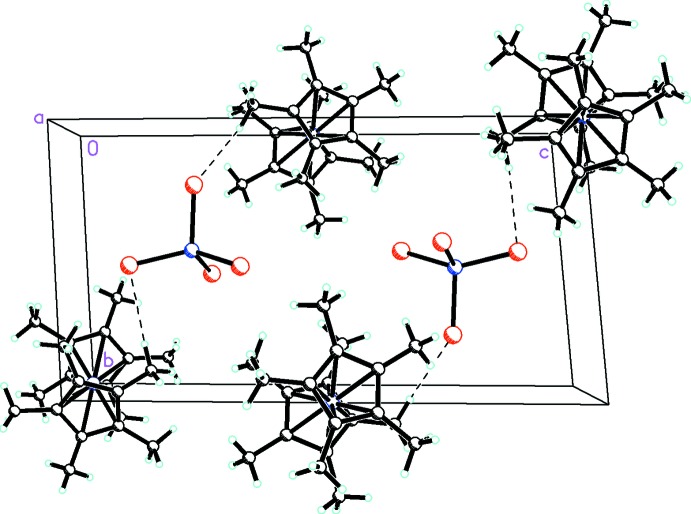
Packing diagram showing the crystal structure and the hydrogen bonding scheme (dashed lines).

## supplementary crystallographic information

### 1.1. Synthesis and crystallization

The starting material Cp*AlBr_2_ was prepared as previously reported (Schormann *et al.*, 2001), and all compounds were handled in an Ar filled dry box or Schlenk glassware. Aluminium tribromide (0.4367 g, 1.63 mmol) was combined with Cp*AlBr_2_ (0.1607 g, 0.50 mmol) in about 10 mL of dry toluene, and NaK_2_ eutectic (0.2002 g, 1.97 mmol) was added. The Kontes valve on the tube was closed and the tube sonicated for 1 day in an ordinary sonic cleaner. Inside the dry box, the mixture was filtered to afford a yellow solution, which was pumped to dryness, affording 0.0619 g of raw product. Recrystallization from heptane by slow evaporation produced crystals of the title compound. ^27^Al-NMR of product [reference: Al(NO_3_)_3_ in HNO_3_ acidified water = 0 ppm]: -114.4 (Cp*_2_Al^+^), 80.4 ppm (AlBr_4_^-^).

### 1.2. Refinement

Crystal data, data collection and structure refinement details are summarized in Table 1. The bromine atoms Br3 and Br4 are disordered over two positions with occupancies of 0.733 (6) and 0.267 (6), respectively. These Br's along with Br1 and Br2 were restrained to be tetra­hedral, *i.e*. Br1/Br2/Br3a/Br4a as one tetra­hedral set and Br1/Br2/Br3b/Br4b as another tetra­hedral set. The H atoms were placed in idealized positions, with CH_3_ groups as rigid groups free to rotate and C—H bond lengths constrained to 0.98 Å.

### 2. Results and discussion

The cluster compound (AlCp*)_4_ is easily made from alkali metal reduction of Cp*Al*X*_2_ (*X*=Cl, Br, I) compounds (Schormann *et al.*, 2001; Minasian & Arnold, 2008), but larger Cp*Al clusters have only been reported from syntheses that start with Al(I) halide clusters, and that starting material requires a difficult and expensive apparatus to synthesize (Vollet *et al.*, 2004, 2005). We attempted to prepare larger clusters by alkali reduction of mixtures of Cp*AlBr_2_ and AlBr_3_ in toluene. The soluble portion of the reaction mixture was recrystallized to afford the colorless title compound (Fig. 1). Other unidentified species with a broad ^27^Al-NMR peak at -20 ppm and a sharp peak at 97 ppm were also present, in addition to Cp*_2_Al ^+^ AlBr_4_^-^.

Deca­methyl­aluminocenium tetra­bromo­aluminate has 1/2 of two Cp*_2_Al^+^ cations in its asymmetric unit, as the two cations are crystallographically independent. As is normal for this cation, the Al atom is linear with the centroids of both rings, with the methyl groups staggered, and the linearity is in this case by symmetry. The two distinct deca­methyl­aluminocenium cations are tilted with respect to each other as the dihedral angle between the ring planes of the two cations is 47.51°. This is in contrast with the AlCl_4_^-^ analog (Schurko *et al.*, 2002; Macdonald *et al.*, 2008), which has two whole molecules in its unit cell, dihedral angles of 43.45-43.98° between the various Cp* ring planes, and near, but not exact, linearity of the centroids of the Cp* rings and their sandwiched Al atoms. The Cp*_2_Al^+^ cation has also been reported in other compounds (Üffing *et al.*, 1999; Kruczyński *et al.*, 2012; Vollet *et al.*, 2006; Burns *et al.*, 1999). The AlBr_4_^-^ anion shows disorder in two of the bromine atoms, and there appears to be weak hydrogen bonding inter­actions between some of the methyl protons and Br1 and Br3 (Fig. 2). While the angle between the undisordered Br atoms and Al3 (Br1—Al3—Br2) of 109.71 (4)° is close to the ideal tetra­hedral angle of 109.5°, not much can really be said about the angles to the disordered Br atoms except that the geometry is approximately tetra­hedral.

Based on other reports of Cp*_2_Al^+^ formation (Dohmeier *et al.*, 1993; Üffing *et al.*, 1998), we presume that inter­actions between (AlCp*)_4_ and AlBr_3_ is probably responsible for the formation of the title compound. A repeat of the same reduction reaction at 0 °C resulted in (AlCp*)_4_ as the only soluble Al-containing product, based on its ^27^Al-NMR peak at -80.7 ppm.

### Crystal data

**Table:**

[Al(C_10_H_15_)_2_][AlBr_4_]	*Z* = 2
*M**_r_* = 644.04	*F*(000) = 632
Triclinic, *P*1	*D*_x_ = 1.703 Mg m^−^^3^
Hall symbol: -P 1	Mo *K*α radiation, λ = 0.71073 Å
*a* = 7.8152 (4) Å	Cell parameters from 5355 reflections
*b* = 9.2949 (4) Å	θ = 3.1–40.9°
*c* = 17.5420 (8) Å	µ = 6.48 mm^−^^1^
α = 85.394 (4)°	*T* = 123 K
β = 85.107 (4)°	Prism, colorless
γ = 82.659 (4)°	0.75 × 0.28 × 0.14 mm
*V* = 1256.12 (10) Å^3^	

### Data collection

**Table:**

Agilent Xcalibur (Ruby, Gemini) diffractometer	9539 independent reflections
Radiation source: Enhance (Mo) X-ray Source	5959 reflections with *I* > 2σ(*I*)
Graphite monochromator	*R*_int_ = 0.038
Detector resolution: 10.5081 pixels mm^-1^	θ_max_ = 33.1°, θ_min_ = 3.1°
ω scans	*h* = −12→9
Absorption correction: multi-scan (*CrysAlis PRO*; Agilent, 2012)	*k* = −14→14
*T*_min_ = 0.116, *T*_max_ = 0.506	*l* = −23→26
19757 measured reflections	

### Refinement

**Table:**

Refinement on *F*^2^	Secondary atom site location: difference Fourier map
Least-squares matrix: full	Hydrogen site location: inferred from neighbouring sites
*R*[*F*^2^ > 2σ(*F*^2^)] = 0.043	H-atom parameters constrained
*wR*(*F*^2^) = 0.072	*w* = 1/[σ^2^(*F*_o_^2^)] where *P* = (*F*_o_^2^ + 2*F*_c_^2^)/3
*S* = 0.91	(Δ/σ)_max_ = 0.001
9539 reflections	Δρ_max_ = 1.58 e Å^−^^3^
256 parameters	Δρ_min_ = −1.29 e Å^−^^3^
24 restraints	Extinction correction: *SHELXL2013* (Sheldrick, 2008), Fc^*^=kFc[1+0.001xFc^2^λ^3^/sin(2θ)]^-1/4^
0 constraints	Extinction coefficient: 0.00092 (12)
Primary atom site location: structure-invariant direct methods	

### Fractional atomic coordinates and isotropic or equivalent isotropic displacement parameters (Å^2^)

**Table:**

	*x*	*y*	*z*	*U*_iso_*/*U*_eq_	Occ. (<1)
Br1	0.96698 (4)	0.21881 (3)	0.25435 (2)	0.03406 (8)	
Br2	0.65528 (4)	0.55941 (4)	0.27080 (2)	0.03666 (9)	
Br3A	0.9871 (7)	0.5256 (8)	0.1151 (4)	0.0541 (4)	0.267 (6)
Br4A	1.0853 (5)	0.5280 (4)	0.3398 (3)	0.0544 (4)	0.267 (6)
Br3B	1.0273 (3)	0.5371 (3)	0.11613 (15)	0.0541 (4)	0.733 (6)
Br4B	1.1204 (2)	0.55802 (18)	0.31528 (14)	0.0544 (4)	0.733 (6)
Al1	0.5000	1.0000	0.0000	0.0211 (2)	
Al2	0.5000	0.0000	0.5000	0.0181 (2)	
Al3	0.93792 (11)	0.46832 (9)	0.24082 (5)	0.02554 (19)	
C1A	0.6158 (4)	1.1583 (3)	0.05506 (15)	0.0267 (6)	
C2A	0.6105 (4)	1.2014 (3)	−0.02423 (15)	0.0251 (6)	
C3A	0.7084 (4)	1.0894 (3)	−0.06667 (15)	0.0243 (6)	
C4A	0.7753 (3)	0.9763 (3)	−0.01345 (15)	0.0247 (6)	
C5A	0.7184 (4)	1.0177 (3)	0.06290 (15)	0.0248 (6)	
C6A	0.5357 (4)	1.2443 (3)	0.12124 (16)	0.0364 (8)	
H6AA	0.6188	1.3054	0.1362	0.055*	
H6AB	0.5048	1.1774	0.1648	0.055*	
H6AC	0.4315	1.3061	0.1057	0.055*	
C7A	0.5193 (4)	1.3404 (3)	−0.05916 (17)	0.0338 (7)	
H7AA	0.6012	1.4122	−0.0698	0.051*	
H7AB	0.4241	1.3781	−0.0233	0.051*	
H7AC	0.4731	1.3213	−0.1071	0.051*	
C8A	0.7364 (4)	1.0949 (3)	−0.15239 (15)	0.0311 (7)	
H8AA	0.8285	1.1551	−0.1694	0.047*	
H8AB	0.6292	1.1368	−0.1750	0.047*	
H8AC	0.7700	0.9962	−0.1688	0.047*	
C9A	0.8866 (4)	0.8369 (3)	−0.03221 (16)	0.0298 (6)	
H9AA	1.0049	0.8411	−0.0187	0.045*	
H9AB	0.8877	0.8244	−0.0872	0.045*	
H9AC	0.8395	0.7547	−0.0029	0.045*	
C10A	0.7629 (4)	0.9341 (3)	0.13688 (16)	0.0320 (7)	
H10D	0.8595	0.9730	0.1570	0.048*	
H10E	0.7962	0.8313	0.1277	0.048*	
H10F	0.6621	0.9434	0.1742	0.048*	
C1B	0.6860 (3)	0.0207 (3)	0.58074 (15)	0.0217 (5)	
C2B	0.6425 (3)	−0.1248 (3)	0.58812 (15)	0.0230 (6)	
C3B	0.6905 (3)	−0.1866 (3)	0.51579 (15)	0.0222 (5)	
C4B	0.7604 (3)	−0.0790 (3)	0.46392 (15)	0.0217 (5)	
C5B	0.7598 (3)	0.0501 (3)	0.50399 (15)	0.0215 (5)	
C6B	0.6614 (4)	0.1239 (3)	0.64341 (16)	0.0324 (7)	
H6BA	0.7571	0.1021	0.6768	0.049*	
H6BB	0.5518	0.1131	0.6736	0.049*	
H6BC	0.6593	0.2240	0.6208	0.049*	
C7B	0.5666 (4)	−0.1986 (3)	0.65955 (16)	0.0315 (7)	
H7BA	0.6585	−0.2338	0.6935	0.047*	
H7BB	0.5110	−0.2810	0.6462	0.047*	
H7BC	0.4805	−0.1295	0.6857	0.047*	
C8B	0.6735 (4)	−0.3398 (3)	0.49793 (18)	0.0327 (7)	
H8BA	0.7824	−0.4019	0.5061	0.049*	
H8BB	0.6470	−0.3405	0.4444	0.049*	
H8BC	0.5800	−0.3767	0.5317	0.049*	
C9B	0.8314 (3)	−0.0988 (3)	0.38248 (15)	0.0290 (6)	
H9BA	0.9574	−0.1228	0.3809	0.043*	
H9BB	0.8025	−0.0085	0.3509	0.043*	
H9BC	0.7804	−0.1778	0.3626	0.043*	
C10B	0.8249 (4)	0.1886 (3)	0.47134 (17)	0.0288 (6)	
H10A	0.9509	0.1721	0.4615	0.043*	
H10B	0.7944	0.2637	0.5080	0.043*	
H10C	0.7719	0.2205	0.4232	0.043*	

### Atomic displacement parameters (Å^2^)

**Table:**

	*U*^11^	*U*^22^	*U*^33^	*U*^12^	*U*^13^	*U*^23^
Br1	0.0522 (2)	0.02018 (14)	0.02845 (16)	−0.00475 (13)	0.00479 (14)	−0.00135 (11)
Br2	0.02744 (15)	0.04200 (19)	0.03929 (19)	0.00291 (14)	0.00066 (13)	−0.01010 (14)
Br3A	0.0598 (9)	0.0352 (4)	0.0534 (3)	0.0103 (6)	0.0309 (7)	0.0184 (3)
Br4A	0.0404 (5)	0.0323 (5)	0.0970 (10)	−0.0003 (4)	−0.0318 (6)	−0.0236 (6)
Br3B	0.0598 (9)	0.0352 (4)	0.0534 (3)	0.0103 (6)	0.0309 (7)	0.0184 (3)
Br4B	0.0404 (5)	0.0323 (5)	0.0970 (10)	−0.0003 (4)	−0.0318 (6)	−0.0236 (6)
Al1	0.0283 (6)	0.0169 (5)	0.0179 (6)	−0.0012 (5)	−0.0026 (4)	−0.0020 (4)
Al2	0.0161 (5)	0.0206 (5)	0.0172 (5)	−0.0002 (4)	−0.0017 (4)	−0.0013 (4)
Al3	0.0259 (4)	0.0191 (4)	0.0315 (5)	−0.0011 (3)	−0.0023 (3)	−0.0029 (3)
C1A	0.0390 (16)	0.0183 (12)	0.0230 (14)	−0.0020 (12)	−0.0037 (12)	−0.0032 (11)
C2A	0.0374 (15)	0.0168 (12)	0.0210 (13)	−0.0038 (11)	−0.0037 (11)	0.0016 (10)
C3A	0.0314 (14)	0.0207 (13)	0.0211 (13)	−0.0052 (11)	−0.0003 (11)	−0.0015 (10)
C4A	0.0288 (14)	0.0223 (13)	0.0228 (14)	−0.0035 (11)	−0.0005 (11)	−0.0008 (11)
C5A	0.0327 (14)	0.0227 (13)	0.0197 (13)	−0.0026 (12)	−0.0070 (11)	−0.0008 (10)
C6A	0.057 (2)	0.0279 (15)	0.0243 (15)	0.0056 (15)	−0.0104 (14)	−0.0110 (12)
C7A	0.0491 (19)	0.0212 (14)	0.0313 (16)	−0.0014 (14)	−0.0102 (14)	−0.0004 (12)
C8A	0.0373 (16)	0.0341 (16)	0.0209 (14)	−0.0047 (13)	0.0012 (12)	0.0004 (12)
C9A	0.0322 (15)	0.0277 (15)	0.0282 (15)	0.0008 (12)	0.0004 (12)	−0.0043 (12)
C10A	0.0412 (17)	0.0301 (15)	0.0245 (15)	0.0008 (14)	−0.0085 (13)	−0.0016 (12)
C1B	0.0197 (12)	0.0250 (13)	0.0206 (13)	−0.0006 (11)	−0.0047 (10)	−0.0027 (10)
C2B	0.0208 (12)	0.0279 (14)	0.0188 (13)	0.0015 (11)	−0.0022 (10)	0.0015 (10)
C3B	0.0196 (12)	0.0218 (13)	0.0237 (13)	0.0026 (10)	−0.0007 (10)	−0.0009 (10)
C4B	0.0189 (11)	0.0248 (13)	0.0208 (13)	−0.0001 (10)	0.0002 (10)	−0.0040 (10)
C5B	0.0185 (11)	0.0247 (13)	0.0212 (13)	−0.0016 (10)	−0.0021 (10)	−0.0009 (10)
C6B	0.0347 (15)	0.0430 (18)	0.0207 (14)	−0.0030 (14)	−0.0037 (12)	−0.0103 (13)
C7B	0.0306 (15)	0.0385 (17)	0.0219 (14)	−0.0004 (13)	0.0008 (12)	0.0093 (12)
C8B	0.0371 (16)	0.0222 (14)	0.0375 (17)	−0.0003 (13)	−0.0019 (13)	−0.0007 (12)
C9B	0.0276 (14)	0.0335 (16)	0.0241 (14)	0.0008 (12)	0.0050 (11)	−0.0058 (12)
C10B	0.0268 (14)	0.0289 (15)	0.0308 (16)	−0.0069 (12)	−0.0001 (12)	−0.0003 (12)

### Geometric parameters (Å, º)

**Table:**

Br1—Al3	2.2963 (9)	C6A—H6AC	0.9800
Br2—Al3	2.2952 (8)	C7A—H7AA	0.9800
Br3A—Al3	2.239 (7)	C7A—H7AB	0.9800
Br4A—Al3	2.304 (4)	C7A—H7AC	0.9800
Br3B—Al3	2.304 (2)	C8A—H8AA	0.9800
Br4B—Al3	2.2869 (14)	C8A—H8AB	0.9800
Al1—C4A	2.129 (3)	C8A—H8AC	0.9800
Al1—C4A^i^	2.129 (3)	C9A—H9AA	0.9800
Al1—C3A	2.139 (3)	C9A—H9AB	0.9800
Al1—C3A^i^	2.139 (3)	C9A—H9AC	0.9800
Al1—C5A	2.140 (3)	C10A—H10D	0.9800
Al1—C5A^i^	2.140 (3)	C10A—H10E	0.9800
Al1—C1A^i^	2.153 (3)	C10A—H10F	0.9800
Al1—C1A	2.153 (3)	C1B—C2B	1.429 (4)
Al1—C2A	2.157 (3)	C1B—C5B	1.438 (4)
Al1—C2A^i^	2.157 (3)	C1B—C6B	1.501 (3)
Al2—C4B^ii^	2.134 (2)	C2B—C3B	1.436 (3)
Al2—C4B	2.134 (2)	C2B—C7B	1.494 (4)
Al2—C5B^ii^	2.148 (3)	C3B—C4B	1.426 (4)
Al2—C5B	2.148 (3)	C3B—C8B	1.507 (4)
Al2—C1B	2.150 (3)	C4B—C5B	1.438 (3)
Al2—C1B^ii^	2.150 (3)	C4B—C9B	1.504 (3)
Al2—C3B^ii^	2.152 (2)	C5B—C10B	1.497 (4)
Al2—C3B	2.152 (2)	C6B—H6BA	0.9800
Al2—C2B^ii^	2.157 (3)	C6B—H6BB	0.9800
Al2—C2B	2.157 (3)	C6B—H6BC	0.9800
C1A—C2A	1.419 (4)	C7B—H7BA	0.9800
C1A—C5A	1.447 (3)	C7B—H7BB	0.9800
C1A—C6A	1.508 (3)	C7B—H7BC	0.9800
C2A—C3A	1.431 (3)	C8B—H8BA	0.9800
C2A—C7A	1.505 (4)	C8B—H8BB	0.9800
C3A—C4A	1.427 (4)	C8B—H8BC	0.9800
C3A—C8A	1.499 (4)	C9B—H9BA	0.9800
C4A—C5A	1.441 (3)	C9B—H9BB	0.9800
C4A—C9A	1.508 (3)	C9B—H9BC	0.9800
C5A—C10A	1.502 (4)	C10B—H10A	0.9800
C6A—H6AA	0.9800	C10B—H10B	0.9800
C6A—H6AB	0.9800	C10B—H10C	0.9800
			
C4A—Al1—C4A^i^	180.0	C3A—C2A—Al1	69.87 (16)
C4A—Al1—C3A	39.05 (10)	C7A—C2A—Al1	125.4 (2)
C4A^i^—Al1—C3A	140.95 (10)	C4A—C3A—C2A	108.3 (2)
C4A—Al1—C3A^i^	140.95 (10)	C4A—C3A—C8A	127.0 (2)
C4A^i^—Al1—C3A^i^	39.05 (10)	C2A—C3A—C8A	124.7 (2)
C3A—Al1—C3A^i^	180.0	C4A—C3A—Al1	70.10 (17)
C4A—Al1—C5A	39.46 (10)	C2A—C3A—Al1	71.23 (16)
C4A^i^—Al1—C5A	140.54 (10)	C8A—C3A—Al1	125.4 (2)
C3A—Al1—C5A	65.71 (11)	C3A—C4A—C5A	108.1 (2)
C3A^i^—Al1—C5A	114.29 (11)	C3A—C4A—C9A	126.9 (2)
C4A—Al1—C5A^i^	140.54 (10)	C5A—C4A—C9A	125.0 (2)
C4A^i^—Al1—C5A^i^	39.46 (10)	C3A—C4A—Al1	70.85 (16)
C3A—Al1—C5A^i^	114.29 (11)	C5A—C4A—Al1	70.68 (15)
C3A^i^—Al1—C5A^i^	65.71 (11)	C9A—C4A—Al1	124.2 (2)
C5A—Al1—C5A^i^	180.0	C4A—C5A—C1A	107.1 (2)
C4A—Al1—C1A^i^	114.31 (10)	C4A—C5A—C10A	126.7 (2)
C4A^i^—Al1—C1A^i^	65.69 (10)	C1A—C5A—C10A	126.2 (2)
C3A—Al1—C1A^i^	114.91 (10)	C4A—C5A—Al1	69.86 (15)
C3A^i^—Al1—C1A^i^	65.09 (10)	C1A—C5A—Al1	70.79 (16)
C5A—Al1—C1A^i^	140.62 (10)	C10A—C5A—Al1	126.4 (2)
C5A^i^—Al1—C1A^i^	39.38 (10)	C1A—C6A—H6AA	109.5
C4A—Al1—C1A	65.69 (10)	C1A—C6A—H6AB	109.5
C4A^i^—Al1—C1A	114.31 (10)	H6AA—C6A—H6AB	109.5
C3A—Al1—C1A	65.09 (10)	C1A—C6A—H6AC	109.5
C3A^i^—Al1—C1A	114.90 (10)	H6AA—C6A—H6AC	109.5
C5A—Al1—C1A	39.38 (10)	H6AB—C6A—H6AC	109.5
C5A^i^—Al1—C1A	140.62 (10)	C2A—C7A—H7AA	109.5
C1A^i^—Al1—C1A	180.00 (12)	C2A—C7A—H7AB	109.5
C4A—Al1—C2A	65.39 (10)	H7AA—C7A—H7AB	109.5
C4A^i^—Al1—C2A	114.61 (10)	C2A—C7A—H7AC	109.5
C3A—Al1—C2A	38.90 (9)	H7AA—C7A—H7AC	109.5
C3A^i^—Al1—C2A	141.10 (9)	H7AB—C7A—H7AC	109.5
C5A—Al1—C2A	65.43 (11)	C3A—C8A—H8AA	109.5
C5A^i^—Al1—C2A	114.57 (11)	C3A—C8A—H8AB	109.5
C1A^i^—Al1—C2A	141.56 (10)	H8AA—C8A—H8AB	109.5
C1A—Al1—C2A	38.44 (10)	C3A—C8A—H8AC	109.5
C4A—Al1—C2A^i^	114.61 (10)	H8AA—C8A—H8AC	109.5
C4A^i^—Al1—C2A^i^	65.39 (10)	H8AB—C8A—H8AC	109.5
C3A—Al1—C2A^i^	141.10 (9)	C4A—C9A—H9AA	109.5
C3A^i^—Al1—C2A^i^	38.90 (9)	C4A—C9A—H9AB	109.5
C5A—Al1—C2A^i^	114.57 (11)	H9AA—C9A—H9AB	109.5
C5A^i^—Al1—C2A^i^	65.43 (11)	C4A—C9A—H9AC	109.5
C1A^i^—Al1—C2A^i^	38.44 (10)	H9AA—C9A—H9AC	109.5
C1A—Al1—C2A^i^	141.56 (10)	H9AB—C9A—H9AC	109.5
C2A—Al1—C2A^i^	180.0	C5A—C10A—H10D	109.5
C4B^ii^—Al2—C4B	180.0	C5A—C10A—H10E	109.5
C4B^ii^—Al2—C5B^ii^	39.25 (9)	H10D—C10A—H10E	109.5
C4B—Al2—C5B^ii^	140.75 (9)	C5A—C10A—H10F	109.5
C4B^ii^—Al2—C5B	140.75 (9)	H10D—C10A—H10F	109.5
C4B—Al2—C5B	39.25 (9)	H10E—C10A—H10F	109.5
C5B^ii^—Al2—C5B	180.00 (14)	C2B—C1B—C5B	108.7 (2)
C4B^ii^—Al2—C1B	114.57 (9)	C2B—C1B—C6B	125.4 (3)
C4B—Al2—C1B	65.43 (9)	C5B—C1B—C6B	126.0 (3)
C5B^ii^—Al2—C1B	140.90 (10)	C2B—C1B—Al2	70.88 (14)
C5B—Al2—C1B	39.10 (10)	C5B—C1B—Al2	70.40 (14)
C4B^ii^—Al2—C1B^ii^	65.43 (9)	C6B—C1B—Al2	125.53 (17)
C4B—Al2—C1B^ii^	114.57 (9)	C1B—C2B—C3B	107.5 (2)
C5B^ii^—Al2—C1B^ii^	39.10 (10)	C1B—C2B—C7B	125.5 (2)
C5B—Al2—C1B^ii^	140.90 (10)	C3B—C2B—C7B	127.0 (3)
C1B—Al2—C1B^ii^	180.0	C1B—C2B—Al2	70.37 (14)
C4B^ii^—Al2—C3B^ii^	38.87 (10)	C3B—C2B—Al2	70.39 (15)
C4B—Al2—C3B^ii^	141.13 (10)	C7B—C2B—Al2	126.15 (18)
C5B^ii^—Al2—C3B^ii^	65.33 (10)	C4B—C3B—C2B	108.4 (2)
C5B—Al2—C3B^ii^	114.67 (10)	C4B—C3B—C8B	125.7 (2)
C1B—Al2—C3B^ii^	115.04 (9)	C2B—C3B—C8B	126.0 (3)
C1B^ii^—Al2—C3B^ii^	64.96 (9)	C4B—C3B—Al2	69.87 (14)
C4B^ii^—Al2—C3B	141.13 (10)	C2B—C3B—Al2	70.69 (13)
C4B—Al2—C3B	38.87 (10)	C8B—C3B—Al2	126.13 (19)
C5B^ii^—Al2—C3B	114.67 (10)	C3B—C4B—C5B	108.3 (2)
C5B—Al2—C3B	65.33 (10)	C3B—C4B—C9B	126.2 (2)
C1B—Al2—C3B	64.96 (9)	C5B—C4B—C9B	125.5 (3)
C1B^ii^—Al2—C3B	115.04 (9)	C3B—C4B—Al2	71.26 (13)
C3B^ii^—Al2—C3B	180.0	C5B—C4B—Al2	70.91 (13)
C4B^ii^—Al2—C2B^ii^	65.48 (10)	C9B—C4B—Al2	125.92 (18)
C4B—Al2—C2B^ii^	114.52 (10)	C4B—C5B—C1B	107.2 (2)
C5B^ii^—Al2—C2B^ii^	65.51 (11)	C4B—C5B—C10B	126.0 (2)
C5B—Al2—C2B^ii^	114.49 (11)	C1B—C5B—C10B	126.8 (2)
C1B—Al2—C2B^ii^	141.24 (10)	C4B—C5B—Al2	69.84 (15)
C1B^ii^—Al2—C2B^ii^	38.76 (10)	C1B—C5B—Al2	70.50 (15)
C3B^ii^—Al2—C2B^ii^	38.92 (9)	C10B—C5B—Al2	125.03 (17)
C3B—Al2—C2B^ii^	141.08 (9)	C1B—C6B—H6BA	109.5
C4B^ii^—Al2—C2B	114.52 (10)	C1B—C6B—H6BB	109.5
C4B—Al2—C2B	65.48 (10)	H6BA—C6B—H6BB	109.5
C5B^ii^—Al2—C2B	114.49 (11)	C1B—C6B—H6BC	109.5
C5B—Al2—C2B	65.51 (11)	H6BA—C6B—H6BC	109.5
C1B—Al2—C2B	38.76 (10)	H6BB—C6B—H6BC	109.5
C1B^ii^—Al2—C2B	141.24 (10)	C2B—C7B—H7BA	109.5
C3B^ii^—Al2—C2B	141.08 (9)	C2B—C7B—H7BB	109.5
C3B—Al2—C2B	38.92 (9)	H7BA—C7B—H7BB	109.5
C2B^ii^—Al2—C2B	180.00 (10)	C2B—C7B—H7BC	109.5
Br3A—Al3—Br2	105.36 (15)	H7BA—C7B—H7BC	109.5
Br4B—Al3—Br2	111.17 (5)	H7BB—C7B—H7BC	109.5
Br3A—Al3—Br1	105.60 (19)	C3B—C8B—H8BA	109.5
Br4B—Al3—Br1	110.88 (6)	C3B—C8B—H8BB	109.5
Br2—Al3—Br1	109.71 (4)	H8BA—C8B—H8BB	109.5
Br3A—Al3—Br4A	127.9 (3)	C3B—C8B—H8BC	109.5
Br2—Al3—Br4A	104.55 (10)	H8BA—C8B—H8BC	109.5
Br1—Al3—Br4A	103.04 (10)	H8BB—C8B—H8BC	109.5
Br4B—Al3—Br3B	105.47 (14)	C4B—C9B—H9BA	109.5
Br2—Al3—Br3B	111.46 (7)	C4B—C9B—H9BB	109.5
Br1—Al3—Br3B	108.05 (7)	H9BA—C9B—H9BB	109.5
C2A—C1A—C5A	108.3 (2)	C4B—C9B—H9BC	109.5
C2A—C1A—C6A	127.0 (2)	H9BA—C9B—H9BC	109.5
C5A—C1A—C6A	124.7 (2)	H9BB—C9B—H9BC	109.5
C2A—C1A—Al1	70.94 (15)	C5B—C10B—H10A	109.5
C5A—C1A—Al1	69.83 (15)	C5B—C10B—H10B	109.5
C6A—C1A—Al1	126.8 (2)	H10A—C10B—H10B	109.5
C1A—C2A—C3A	108.3 (2)	C5B—C10B—H10C	109.5
C1A—C2A—C7A	126.8 (2)	H10A—C10B—H10C	109.5
C3A—C2A—C7A	125.0 (2)	H10B—C10B—H10C	109.5
C1A—C2A—Al1	70.62 (17)		
			
C5A—C1A—C2A—C3A	0.1 (3)	C5B—C1B—C2B—C3B	0.4 (3)
C6A—C1A—C2A—C3A	177.9 (3)	C6B—C1B—C2B—C3B	−178.6 (2)
Al1—C1A—C2A—C3A	−60.0 (2)	Al2—C1B—C2B—C3B	60.89 (17)
C5A—C1A—C2A—C7A	−179.7 (3)	C5B—C1B—C2B—C7B	178.5 (2)
C6A—C1A—C2A—C7A	−1.9 (5)	C6B—C1B—C2B—C7B	−0.5 (4)
Al1—C1A—C2A—C7A	120.2 (3)	Al2—C1B—C2B—C7B	−121.0 (2)
C5A—C1A—C2A—Al1	60.1 (2)	C5B—C1B—C2B—Al2	−60.53 (17)
C6A—C1A—C2A—Al1	−122.2 (3)	C6B—C1B—C2B—Al2	120.5 (2)
C1A—C2A—C3A—C4A	−0.2 (3)	C1B—C2B—C3B—C4B	−0.9 (3)
C7A—C2A—C3A—C4A	179.6 (3)	C7B—C2B—C3B—C4B	−179.0 (2)
Al1—C2A—C3A—C4A	−60.6 (2)	Al2—C2B—C3B—C4B	59.97 (17)
C1A—C2A—C3A—C8A	−179.0 (3)	C1B—C2B—C3B—C8B	177.9 (2)
C7A—C2A—C3A—C8A	0.8 (5)	C7B—C2B—C3B—C8B	−0.1 (4)
Al1—C2A—C3A—C8A	120.5 (3)	Al2—C2B—C3B—C8B	−121.2 (3)
C1A—C2A—C3A—Al1	60.5 (2)	C1B—C2B—C3B—Al2	−60.88 (16)
C7A—C2A—C3A—Al1	−119.7 (3)	C7B—C2B—C3B—Al2	121.0 (3)
C2A—C3A—C4A—C5A	0.1 (3)	C2B—C3B—C4B—C5B	1.1 (3)
C8A—C3A—C4A—C5A	178.9 (3)	C8B—C3B—C4B—C5B	−177.7 (2)
Al1—C3A—C4A—C5A	−61.2 (2)	Al2—C3B—C4B—C5B	61.60 (16)
C2A—C3A—C4A—C9A	−179.7 (3)	C2B—C3B—C4B—C9B	178.2 (2)
C8A—C3A—C4A—C9A	−0.9 (5)	C8B—C3B—C4B—C9B	−0.6 (4)
Al1—C3A—C4A—C9A	118.9 (3)	Al2—C3B—C4B—C9B	−121.3 (3)
C2A—C3A—C4A—Al1	61.3 (2)	C2B—C3B—C4B—Al2	−60.49 (17)
C8A—C3A—C4A—Al1	−119.9 (3)	C8B—C3B—C4B—Al2	120.7 (3)
C3A—C4A—C5A—C1A	−0.1 (3)	C3B—C4B—C5B—C1B	−0.9 (3)
C9A—C4A—C5A—C1A	179.8 (3)	C9B—C4B—C5B—C1B	−178.0 (2)
Al1—C4A—C5A—C1A	−61.4 (2)	Al2—C4B—C5B—C1B	60.94 (17)
C3A—C4A—C5A—C10A	−177.8 (3)	C3B—C4B—C5B—C10B	178.9 (2)
C9A—C4A—C5A—C10A	2.1 (5)	C9B—C4B—C5B—C10B	1.8 (4)
Al1—C4A—C5A—C10A	120.9 (3)	Al2—C4B—C5B—C10B	−119.3 (2)
C3A—C4A—C5A—Al1	61.3 (2)	C3B—C4B—C5B—Al2	−61.82 (17)
C9A—C4A—C5A—Al1	−118.8 (3)	C9B—C4B—C5B—Al2	121.0 (3)
C2A—C1A—C5A—C4A	0.0 (3)	C2B—C1B—C5B—C4B	0.3 (3)
C6A—C1A—C5A—C4A	−177.8 (3)	C6B—C1B—C5B—C4B	179.2 (2)
Al1—C1A—C5A—C4A	60.76 (19)	Al2—C1B—C5B—C4B	−60.52 (17)
C2A—C1A—C5A—C10A	177.7 (3)	C2B—C1B—C5B—C10B	−179.5 (2)
C6A—C1A—C5A—C10A	−0.1 (5)	C6B—C1B—C5B—C10B	−0.5 (4)
Al1—C1A—C5A—C10A	−121.5 (3)	Al2—C1B—C5B—C10B	119.7 (2)
C2A—C1A—C5A—Al1	−60.8 (2)	C2B—C1B—C5B—Al2	60.83 (17)
C6A—C1A—C5A—Al1	121.4 (3)	C6B—C1B—C5B—Al2	−120.2 (3)

Symmetry codes: (i) −*x*+1, −*y*+2, −*z*; (ii) −*x*+1, −*y*, −*z*+1.

### Hydrogen-bond geometry (Å, º)

**Table:**

*D*—H···*A*	*D*—H	H···*A*	*D*···*A*	*D*—H···*A*
C7*A*—H7*AA*···Br3*B*^iii^	0.98	3.03	3.886 (4)	146
C9*A*—H9*AC*···Br3*A*	0.98	3.04	3.773 (8)	133
C10*A*—H10*E*···Br3*A*	0.98	3.05	4.007 (7)	165
C9*B*—H9*BB*···Br1	0.98	2.95	3.770 (3)	141

Symmetry code: (iii) −*x*+2, −*y*+2, −*z*.
